# Module-Based Polyketide Synthase Engineering for *de Novo* Polyketide Biosynthesis

**DOI:** 10.1021/acssynbio.3c00282

**Published:** 2023-10-23

**Authors:** Alberto A. Nava, Jacob Roberts, Robert W. Haushalter, Zilong Wang, Jay D. Keasling

**Affiliations:** † Joint BioEnergy Institute, Lawrence Berkeley National Laboratory, Emeryville, California 94608, United States; ‡ Biological Systems and Engineering Division, Lawrence Berkeley National Laboratory, Berkeley, California 94720, United States; § Department of Chemical and Biomolecular Engineering, University of California, Berkeley, Berkeley, California 94720, United States; ∥ Department of Bioengineering, University of California, Berkeley, Berkeley, California 94720, United States; ⊥ Center for Synthetic Biochemistry, Shenzhen Institutes for Advanced Technologies, Shenzhen 518055, P.R. China; # The Novo Nordisk Foundation Center for Biosustainability, Technical University Denmark, Kemitorvet, Building 220, Kongens Lyngby 2800, Denmark

**Keywords:** polyketide synthases, structural modeling, retrobiosynthesis, protein engineering

## Abstract

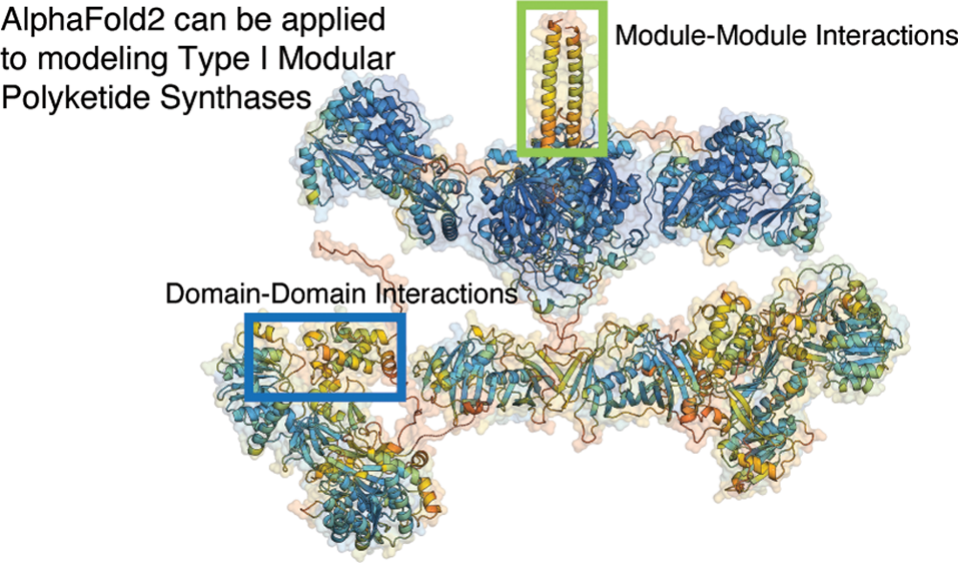

Polyketide retrobiosynthesis, where the biosynthetic pathway of a given polyketide can be reversibly engineered due to the colinearity of the polyketide synthase (PKS) structure and function, has the potential to produce millions of organic molecules. Mixing and matching modules from natural PKSs is one of the routes to produce many of these molecules. Evolutionary analysis of PKSs suggests that traditionally used module boundaries may not lead to the most productive hybrid PKSs and that new boundaries around and within the ketosynthase domain may be more active when constructing hybrid PKSs. As this is still a nascent area of research, the generality of these design principles based on existing engineering efforts remains inconclusive. Recent advances in structural modeling and synthetic biology present an opportunity to accelerate PKS engineering by re-evaluating insights gained from previous engineering efforts with cutting edge tools.

## Introduction

Retrosynthesis is the concept of designing synthetic organic chemistry routes by working backward from the final product to define a series of achievable reactions from simpler building blocks.^1^ Similarly, retrobiosynthesis is the application of a similar concept with the addition of enzyme-catalyzed chemical reactions.^2^ Type I polyketide synthases (PKSs) have been heralded as a potential foundation for retrobiosynthesis since their first elucidation as modular enzymatic assembly lines in the early 1990s.^3,4^ The polyketides produced by PKSs are a diverse class of natural products with extensive bioactivities including antibacterial (e.g., erythromycin), antifungal (e.g., amphotericin B), and anticancer (e.g., epothilone) properties with agricultural and medicinal applications.^5^ Moreover, the stereochemically rich, highly functionalized cores of these compounds pose significant obstacles for synthetic chemists. Thus, the interest and potential in engineering PKSs is based on the valuable activities of these complex compounds, and their colinear biosynthetic logic, meaning that the chemical structure of the compounds produced can be directly inferred by the order and type of the enzymes in the pathway.

PKSs are composed of modules that act as part of an assembly line carrying out stepwise decarboxylative Claisen condensation reactions of acyl-CoA building blocks.^6^ The mechanism of polyketide elongation has long been compared to fatty acid synthesis^7,8^ in that an acyl-CoA extender unit is first primed by an acyl transferase (AT) onto its cognate acyl carrier protein (ACP) before undergoing a decarboxylative Claisen condensation reaction with the polyketide chain tethered to a β-keto synthase (KS). The polyketide β-carbon is then optionally reduced by a NADPH-dependent ketoreductase (KR) to a hydroxyl group, then dehydrated by a dehydratase (DH) to an enoyl group, and finally reduced to a fully saturated β-carbon by an enoyl reductase (ER). After undergoing elongation and reduction, the polyketide chain is shuffled by the ACP to the KS domain of the downstream module where the assembly line continues with the condensation of a new extender unit. At the end of the assembly line, the most common termination domain is a thioesterase (TE), which either hydrolyzes the linear product off the PKS or catalyzes a cyclization reaction.^9−11^ The regio- and stereochemistry of each ketide building block is determined by the combination of conserved catalytic motifs in the KS, KR, DH, and ER.^12^ The structure of the final ketide product can be inferred from the order in which these modules interact, which is directed by both protein–protein interactions and substrate specificity of each domain. Therefore, there is a direct relationship between the DNA sequence of the biosynthetic cluster and the structure of the molecule produced.

PKSs represent an attractive avenue to the access of truly complex carboxylic molecules, and their unique organization has prompted numerous engineering efforts by mutating or shuffling catalytic parts for the biosynthesis of novel products. They have been successfully engineered to produce numerous interesting bioproducts including potential therapeutics,^13^ polymer precursors,^14^ specialty chemicals,^15^ and biofuels.^16^ Nevertheless, many engineered PKSs suffer from reduced catalytic activities.^9^ Two primary overarching issues have prevented PKSs from reaching their full potential: neither the protein–protein interactions nor the substrate–protein interactions within PKSs are well characterized.^10,17−19^ However, recent structural,^20,21^ evolutionary,^22,23^ biochemical,^24^ and metabolic engineering^16^ studies suggest that the traditional boundaries used for module–module connections may not provide optimal interactions between modules, and that there are alternative boundaries that may boost success rates.^24,25^ Furthermore, recent progress in the field of machine learning has enabled impressive models in a diverse range of fields including protein structure prediction. The release of Alphafold2^26^ and RoseTTAFold^27^ for protein structure prediction has prompted a swath of studies investigating complex macromolecular phenomena including protein complex analyses.^28^ In this perspective, we discuss how previous module-based PKS engineering efforts can be re-evaluated in the context of modern structural modeling and how the insights gained may initiate a new generation of PKS design principles.

## Module-Based Polyketide Synthase Engineering

**Figure 1:**
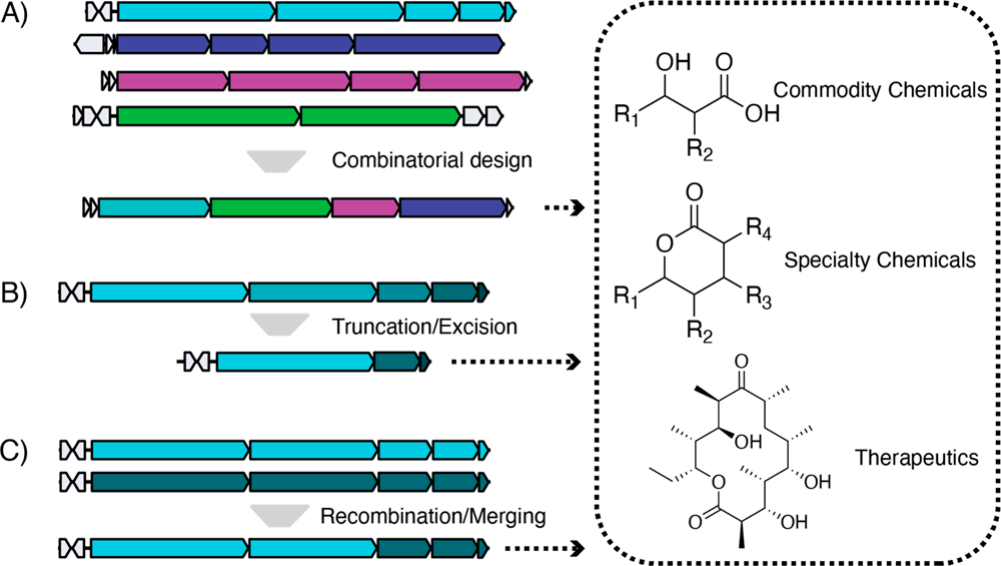
Module-based PKS engineering. (A) Original ideas for a PKS retrobiosynthesis platform involved recombining parts from known PKS biosynthetic gene clusters (BGCs) for the purpose of producing novel molecules. The colors of the genes signify that PKS assembly lines are being composed by a combinatorial library of individual parts (e.g., subunits, modules). (B) Evolutionary guided PKS engineering involves modifying an individual PKS BGC by excision of modules based on recombination principles. The goal being to introduce as few non-native protein–protein interactions as possible. (C) Strategy of recombining two homologous BGCs to produce non-native products. The goal in this strategy is to identify PKS BGCs that allow you to make as few engineering changes as possible in order to achieve a desired final product. On the right side of the figure are a few diverse applications of polyketide molecules: 3-hydroxy acids as commodity chemicals, δ-lactones as specialty chemicals, macrolactones as therapeutics (6-deoxyerythronolide B).

The natural homology existing between PKS modules from different biosynthetic gene clusters (BGCs) within diverse organisms sparked one of the most ambitious goals of PKS engineering, the lego-ization of PKSs. The lego-ization of PKSs involves the combinatorial expression of PKS parts for the biosynthesis of novel bioproducts (Figure 1A).^30,31^ Demonstrating the inherent promiscuity with PKS parts, Menzella et al. successfully biosynthesized over a dozen novel triketide lactones by recombining dozens of PKS modules.^31^ However, the dramatically reduced product titers from most of their engineered constructs showed that there are unknown fundamental principles that govern the interactions between PKS modules.

Subsequent studies that investigated the KS domain from downstream modules revealed that the KS domain plays a critical role in the interactions between chimeric modules, and that swapping the KS can, in some cases, resuscitate inactive interactions.^32^ Though the failures in KS-catalyzed chain extension are presumably multifaceted and still have not been logically resolved, the gatekeeping behavior of KSs has been supported by in-depth biochemical^33^ and evolutionary analyses.^22,23^ The evolutionary analyses provide evidence that KS domains more strongly coevolve with upstream domains, suggesting that an alternative definition of a PKS module would have the KS domain as the last domain of a PKS module instead of the first. This alternative definition of a PKS module (starting at AT and ending after KS) is known as the PKS exchange unit (XU),^34^ analogous to the definition used in non-ribosomal peptide synthetases (NRPSs).^35,36^

This XU model contrasts with the genetic organization of the domains within open reading frames but is logical given that the structural characteristics of a substrate entering a KS active site are determined by the AT and reducing domains. The biochemical analyses provide evidence that the condensation reaction catalyzed by the KS domain is indeed the rate-limiting step.^37^ Notably, this KS gatekeeping activity is much more prevalent in trans-AT PKS assembly lines than in cis-AT PKS systems and has been used to identify generalizable design principles for such systems.^38−40^ Trans-AT PKSs are differentiated from cis-AT PKSs in that the AT domain, which is responsible for loading an extender unit onto the ACP, is not located within the same module that performs chain elongation. Instead, the AT domain is either found as a separate, standalone enzyme or occasionally within a different module. Though closely related to cis-AT PKSs, the unique architectural nuances of trans-AT PKSs mean that some but not all engineering strategies can be directly generalized between systems.

Insights about the mechanism of PKS diversity have emerged from the observation that the phylogeny of KS domains aligns closely with the host organism phylogeny and that KS domains are most closely related to other KS domains within the same PKS, and more broadly to other KS domains within the same host organism. This suggests a model that PKSs evolved primarily through horizontal gene/BGC transfer, recombination, gene conversion, and genetic drift, rather than through gene duplication.^41−44^ Furthermore, this model implies that many evolutionary means exist to alleviate reduced activity at module boundaries via convergent evolution. It should continue to be a key area of focus to determine what biochemical aspects of PKS evolution can be generalized.

The consideration of these factors has led to some recent evolutionary-inspired PKS engineering strategies (Figure 1B,C). For instance, the Tylosin and Rapamycin PKSs were the target of an accelerated evolution experiment that produced numerous active truncated assembly lines.^13^ Notably, the mutant assembly lines were not generated through targeted *in vitro* cloning, but rather through spontaneous *in vivo* homologous recombination. The authors reported that when modules were deleted from the native PKS, the place where recombination generally occurred was not particularly on either end of the KS but rather throughout the KS domain, AT domain, and the interdomain linker region upstream of the ACP, with a notable hot spot for recombination within the middle of the KS that takes advantage of the high sequence homology between KS domains. The Pikromycin PKS is another significant case study system providing evidence that utilizing XU module boundaries can improve activity of chimeric PKSs.^24,45^ Moreover, *in vivo* homologous recombination engineering strategies have also been applied to the Pikromycin PKS and similarly demonstrate successful recombination throughout the KS and AT domains.^46^ The Aureothin and Neoaureothin PKSs are two homologous BGCs that were successfully engineered to produce non-native products by utilizing XU module boundaries.^47^

The Stambomycin PKS has been subject to one of the most systematic studies of module boundaries to date, where six different approaches to generating a truncated version of the assembly line were applied.^34^ The goal of Su et al. was to engineer a successful interaction between module 13 and module 21 of the Stambomycin PKS.^34^ They compared six strategies for engineering this interaction including the following: swapping the C-terminal docking domain of module 13 for the C-terminal docking domain of module 20 [nonfunctional]; swapping the C-terminal docking domain and performing a mutation in the KS-ACP interface region of the ACP in module 13 [functional]; swapping the KS domain of module 21 for the KS domain of module 14 utilizing XU boundaries [functional]; swapping the KS domain of module 21 for the KS domain of module 14 utilizing the recombination hot spot boundaries [functional]; swapping the KS domain of module 21 for the KS domain of module 14 utilizing XU boundaries and swapping the KS-ACP interface region of the ACP in module 21 for the KS-ACP interface region of the ACP in module 14 [nonfunctional]; and last swapping the KS domain of module 21 for the KS domain of module 14 utilizing XU boundaries and performing a G to D mutation in the ACP of module 21 [functional].^34^ The functional variants were successfully able to produce measurable quantities of truncated final product; notably, the variant that swapped the downstream KS domain using the recombination hot spot junction in the middle of the KS domain achieved the best kinetics.^34^

**Figure 2:**
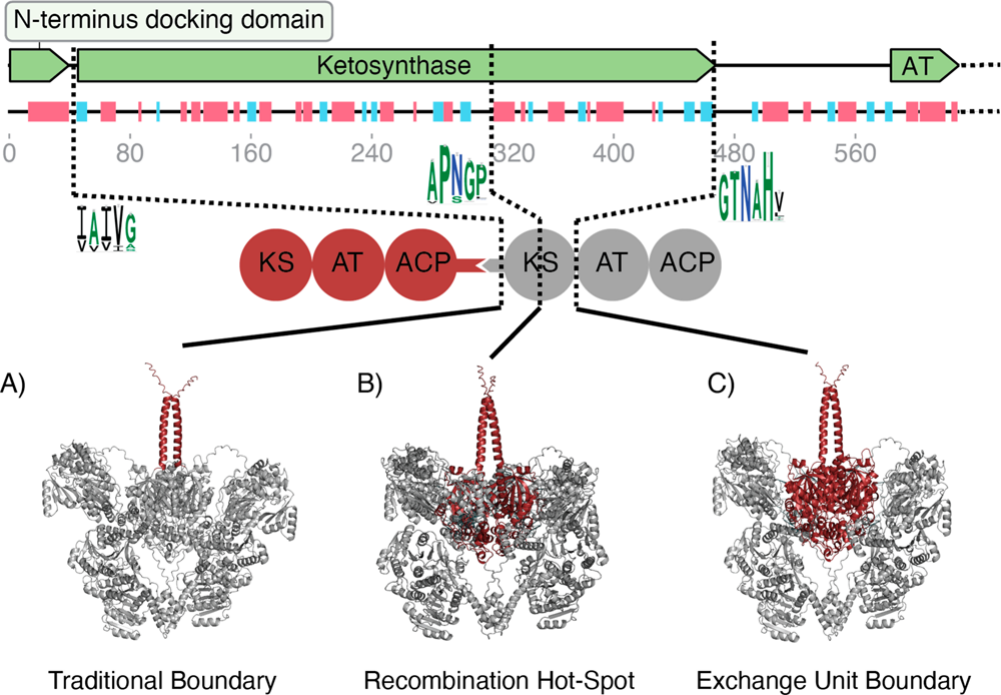
Module junction selected is critical to the solubility and activity of engineered PKSs. There are primarily three junctions that have been explored: (A) the traditional boundary at the start of the KS domain, (B) a recombination hot spot in the middle of the KS domain, and (C) the exchange unit boundary at the end of the KS domain. Shown is Module 21 of the Stambomycin PKS (Sta21) modeled by AlphaFold2 with each module junction (from Su et al.^34^) highlighted in red. Domain annotations within the boundary region are shown at the top of the figure with a secondary structure plot of the KS domain in Sta21 with α-helices as pink-colored boxes and β-sheets as teal-colored boxes. Secondary structure analysis was performed with the PSIPRED server.^29^

Recombination at each of the three junctions (Figure 2, Figure S1) have exhibited some success in engineered systems; however, without a systematic study examining module boundaries in a variety of systems, it is difficult to identify generalizable design principles. One promising approach that has recently been applied for the optimization of AT-swap junctions that could potentially be used for more systematically analyzing module–module interactions involves the use of oligonucleotide pools for generating libraries of junctions, a solubility biosensor for performing a preliminary high throughput screen of high expressing variants, and a kinetic analysis of a diverse subset of the library.^48^ This approach is amenable to high throughput experimentation and could enable more generalizable junctions by systematically characterizing the space of possible junctions. For now, recent evidence (Table 1) suggests that either the XU module boundary or recombination hot spot are more likely to yield active variants, but it is clear that there is not a one-junction-fits-all type of rule and it remains to be determined whether there are alternative junctions which would yield better activity.

**Table 1:** Investigations into PKS Module Boundaries

**Module Boundary**	**Rationale for Engineering**	**Product**	**References**
Traditional boundary	High sequence conservation and convenient restriction sites	Triketide lactones	Menzella et al.^31^
Exchange unit boundary	KS gatekeeping hypothesis	Triketide lactones	Chandran et al.^32^
Recombination hot spot	Homology-directed recombination	Rapalogs	Wlodek et al.^13^
Traditional and exchange unit boundary	Co-evolution analysis; after KS was best	Triketide lactones	Miyazawa et al.^24^
All 3 boundaries	Previous literature; middle of KS was best	Mini-stambomycins	Su et al.^34^

## Polyketide Synthase Dynamics

PKSs are very large and highly dynamic homodimeric proteins, which means that structural characterization of PKS assembly lines was until recently limited to individual domains or didomains. For example, docking domains,^49−51^ AT domains,^52,53^ KS-AT didomains,^54,55^ DH domains,^56,57^ KR-ER didomains,^58^ TE domains,^59,60^ and KR domains^61^ have all been individually characterized. However, the activity of PKS assembly lines is highly dependent on the interactions between domains, which are not able to be completely described from structures of isolated domains. In particular, for the purpose of module-based PKS engineering, there are several structural features that critically determine catalytic activity including the following: (i) the ability of an ACP domain to translocate a polyketide chain to a downstream KS domain, (ii) the ability of a KS domain to catalyze the condensation of a polyketide chain with an extender unit, (iii) and the ability of an ACP domain to translocate a ketide extender unit with an upstream KS domain.

There are several recent reports of larger multidomain structural characterization efforts that reveal insights into how to potentially improve module-based engineering. The first direct description of module–module interactions utilized the ability of small-angle X-ray scattering (SAXS) to capture low-resolution structures of proteins in the Erythromycin PKS without the need for crystallization.^62^ This revealed conformations that active PKS modules undergo; however, many key details about intermodule communication could not be elucidated due to the resolution. Cryogenic electron microscopy (cryo-EM) has additionally been used to capture conformational data about a full-length PKS module in the presence of an upstream ACP, resulting in the hypothesis that there are two distinct active sites within the KS domain for intramodule and intermodule ACP interactions.^63,64^ However, the arch-shaped structure observed in this system is different from previous extended-shaped models and is being further rebutted by recent structural data.^65^ Nonetheless, cryo-EM has been used in tandem with the first high-resolution X-ray crystallographic structure of a full-length PKS module to illuminate many of the dynamic events that must happen for intramodule transacylation and chain extension to occur.^20^ Molecular dynamics simulations have been employed to visualize the small-subunit structural changes that occur as an AT domain accepts an extender substrate.^66^ High-resolution cryo-EM structures of an Erythromycin PKS module further reveal dynamics of how an AT domains’ position relative to its cognate KS domain enable transacylation.^21^ In type II fatty acid synthases, several key residues have been identified at the interaction interface between ACP and KS domains that alter the behavior of substrates in the KS active site,^67,68^ and detailed mechanisms of KS catalytic activity have been elucidated.^69,70^ Though the KS-ACP interface is not identical between type II fatty acid synthases and type I PKSs,^71^ the key interplay observed between the ACP domain and its corresponding AT and KS domains sustains the idea that domain–domain interactions incorporating the ACP are crucial to PKS activity. The essentiality of compatible ACP interactions, in particular with the KS domain of a downstream module, has been corroborated in several reports.^17,33^ The recent improvement in the resolution of cryo-EM has enabled critical perspectives to the structural dynamics of PKS enzymes and should continue to be employed to further elucidate trajectories of catalytic activity, as has been done in the NRPS field.^72^

## Protein Modeling for Polyketide Synthases

The release of AlphaFold2^26,73,74^ and RoseTTAFold^27^ as highly accurate protein structure prediction algorithms based on deep-learning models has enabled numerous impressive protein engineering feats including *de novo* protein design^75^ and proteome-scale protein interaction screening.^28^ Similar deep-learning models have been used to develop MutCompute,^76^ a generalizable protein design algorithm that has successfully been applied to optimize the thermostability, pH tolerance, and kinetics of a PET hydrolase.^77^ Furthermore, the advancement of large language models has led to the development of programs including ESMFold,^78^ a rapid protein structure prediction algorithm, and ProGen,^79^ a generative protein sequence algorithm capable of hallucinating proteins with custom properties. Additional tools have enabled more accessible use to AlphaFold2 including ColabFold^74^ and Foldy.^80^ Though these modern protein modeling algorithms have enabled incredible achievements, challenges remain in the modeling of large proteins (>3000 amino acids) and in high accuracy protein complex modeling. Nonetheless, these modern protein modeling algorithms may have the potential to augment our understanding and ability to engineer PKSs.

**Figure 3:**
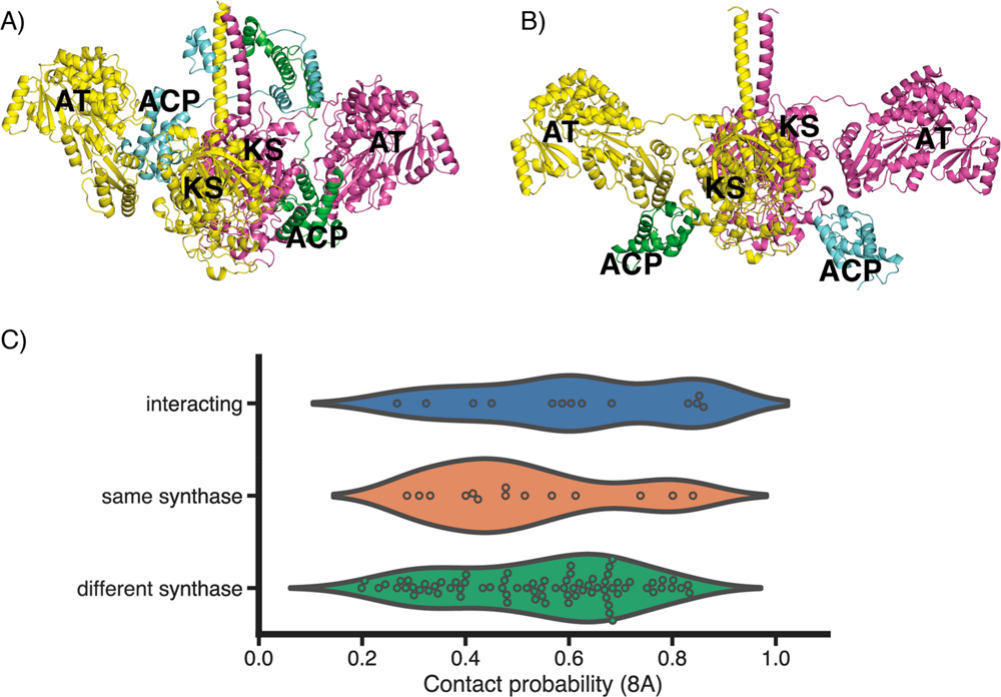
Modern deep-learning protein models applied to PKSs. (A) An AlphaFold2 model of an upstream ACP interacting with a downstream KS-AT with corresponding docking domains. Shown is the Lipomycin PKS Module 1 ACP (green and blue) and Module 2 KS-AT (yellow and pink). (B) An AlphaFold2 model of an ACP interacting with an upstream KS-AT. Shown is the Epothilone PKS Module 7 KS-AT (yellow and pink) and ACP (green and blue). (C) A violin heat map of AlphaFold2 predicted protein–protein contact probabilities (defined as the highest predicted contact probability over all pairs of residues in two protein chains at a threshold of 8 Å^28^) for each of the ACP and KS-AT interactions experimentally characterized in Menzella et al.^31^ Combinations are categorized based on whether they natively interact (blue), whether they are natively in the same PKS BGC (orange), or otherwise from different PKS BGCs (green).

Here we demonstrate the potential applicability of AlphaFold2 to the modeling of PKSs. We have shown that AlphaFold2 is capable of reasonably modeling an entire PKS module (Figure S2) and in some cases demonstrates asymmetric reaction chambers (Figure S3). Each PKS model generated by AlphaFold2 has an extended-shape conformation, and we have not observed any models that have an arch-shaped conformation. This is likely due to the majority of experimentally derived structures of KS-AT domains, which form part of the data set on which AlphaFold2 is trained, being in an extended-shape conformation. An additional attractive feature would be the ability to model multiple modules at the same time. Unfortunately, we have been unable to obtain an AlphaFold2 model of two full-length modules at the same time due to two main reasons: (i) the GPU memory required scales quadratically with the length of the protein leading to out-of-memory errors for protein complexes that are too large and (ii) reducing the memory usage by reducing the size of the multiple sequence alignments input into AlphaFold2 leads to artifacts in the resultant structure such as overlapping chains (Figure S4). Alternatively, we have been able to demonstrate that it is possible to truncate the upstream module to facilitate modeling with a downstream module, thus making it possible to visualize an ACP interacting with both a downstream KS-AT (Figure 3A, Figure S5) and an upstream KS-AT (Figure 3B, Figure S5). In a useful demonstration of how KS-ACP modeling can inform and boost engineering success, Buyachuihan et al. utilized AlphaFold2 models of the Venemycin synthase to improve product yields by optimizing docking domains that take into account the flexibility required at the KS-ACP interface.^81^

These capabilities beg the question of whether structural modeling could be used to predict the likelihood that a given ACP could successfully interact with a given KS domain. We used Alphafold to model the 144 intersubunit ACP and KS-AT interactions experimentally characterized by Menzella et al.^31^ and applied the state-of-the-art protein complex prediction algorithm described by Humphreys et al.^28^ to calculate the probability that a given upstream ACP would successfully interact with a given downstream KS, the hypothesis being that the predicted interaction probability between natively interacting modules would be higher than non-natively interacting modules and that it would correlate with overall production titers. Overall, we observed limited correlation between the predicted interaction probability and the observed production data from Menzella et al.^31^ (Figure S6). This result is not surprising as it is clear that the product titer depends on many factors including non-native substrate tolerance and chimeric protein stability. Also, despite our initial hypothesis that the predicted interaction probability between natively interacting domains would be higher than that between non-natively interacting domains, we see a limited correlation between the predicted interaction probability and whether the interaction between an ACP and KS is natively occurring (Figure 3C). It is possible that the docking domains included in the models, which are non-native to each combination (except for eryM2 ACP and eryM3 KS-AT), lead to discordance in the predictions, or it could also be possible that AlphaFold2 does not have the precision and resolution necessary to accurately differentiate between the highly homologous domains between PKS modules. Ultimately, it is likely that the successful interaction between KS and ACP domains is necessary but not sufficient for productivity and that a more holistic analysis of protein–protein and substrate–protein interactions is necessary for inference. Advancements in the way that the co-evolutionary signal is calculated for colinear multidomain and multisubunit enzyme assembly lines like PKSs should boost the accuracy of these algorithms, by for example using antiSMASH^82^ annotations to update multiple sequence alignments. Additionally, the advancement and incorporation of deep-learning tools capable of handling substrate–protein interactions, such as DiffDock,^83^ should enable more comprehensive modeling of PKS behavior. Lastly, the ability of AlphaFold2 to generate unique stable conformations of PKSs should enable detailed visualizations of PKS dynamics. Ultimately, these tools are at the stage where unique and novel insights can already be obtained, and further improvements will only accelerate what is possible in PKS engineering.

## Outlook

PKSs have shown promise as a retrobiosynthesis platform for production of a wide variety of small molecules. PKS intermodule communication has naturally been a topic of significant interest as it is frequently the bottleneck in engineered systems. PKS collinearity presents an interesting opportunity for machine-learning scientists to test modern protein design strategies on a system with a reduced design space. Modern protein design programs present a complementary opportunity for PKS engineers to obtain optimized PKS designs that take into account evolutionary signals, structural elements, and empirical evidence. The collaboration between these groups should prove beneficial.

## Supporting Information Available

Quality measures of AlphaFold2 model of Stambomycin PKS Module 21 (Figure S1); AlphaFold2 model of Lasalocid PKS Module 14 (Figure S2); AlphaFold2 model of Lipomycin PKS Module 1 demonstrating asymmetric reaction chamber (Figure S3); example of steric artifacts caused by modeling two consecutive PKS modules with AlphaFold2 (Figure S4); quality measures of AlphaFold2 models of KS-AT and ACP interactions used in Figure 3 (Figure S5); graphs of interaction probability between KS and ACP domains (Figure S6) (PDF)
PDB files of AlphaFold2 models (ZIP)
