# Supplementary material for: Module-Based Polyketide Synthase Engineering for de Novo Polyketide Biosynthesis
Source: ACS Synth Biol. 2023 Oct 23;12(11):3148–55. doi: 10.1021/acssynbio.3c00282 (PMC10661043; doi:10.1021/acssynbio.3c00282)
Supplement: Supplementary file 1 — sb3c00282_si_001.pdf [file sb3c00282_si_001.pdf]

Supplementary Information

Module-based polyketide synthase engineering for *de novo* polyketide biosynthesis

Authors:

Alberto A. Nava[1,2,3], Jacob Roberts[1,2,4], Robert W. Haushalter[1,2], Zilong Wang[1,2], Jay D. Keasling[1-6*]

1. Joint BioEnergy Institute, Lawrence Berkeley National Laboratory, Emeryville, CA 94608, USA
2. Biological Systems and Engineering Division, Lawrence Berkeley National Laboratory, Berkeley, California 94720, United States
3. Department of Chemical and Biomolecular Engineering, University of California, Berkeley, Berkeley, CA 94720, USA
4. Department of Bioengineering, University of California, Berkeley, Berkeley, CA 94720, USA
5. Center for Synthetic Biochemistry, Shenzhen Institutes for Advanced Technologies, Shenzhen 518055, P.R. China
6. The Novo Nordisk Foundation Center for Biosustainability, Technical University Denmark, Kemitorvet, Building 220, Kongens Lyngby 2800, Denmark

*Corresponding author: keasling@lbl.gov

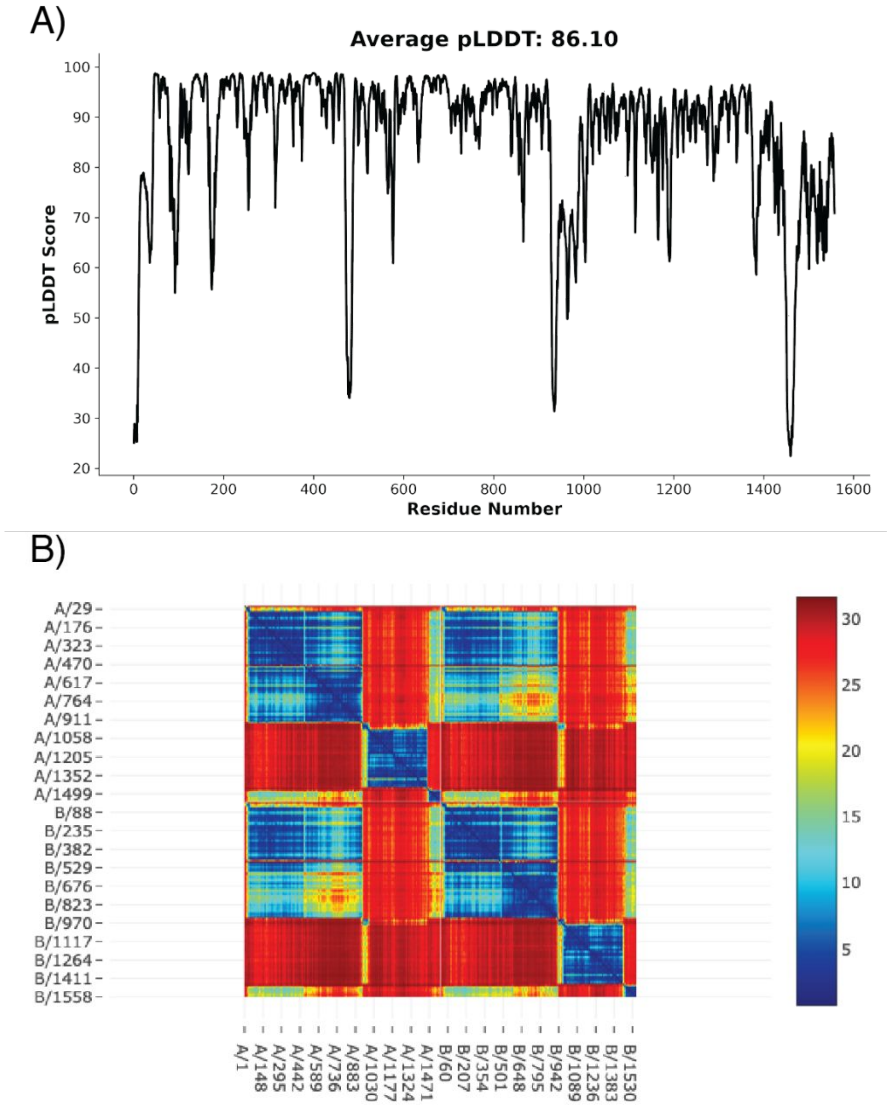

Figure S1. Quality measures of AlphaFold2 model of Stambomycin Module 21 shown in Figure 2 A) AlphaFold2 pLDDT of model chain A by residue number B) AlphaFold2 predicted aligned error of model

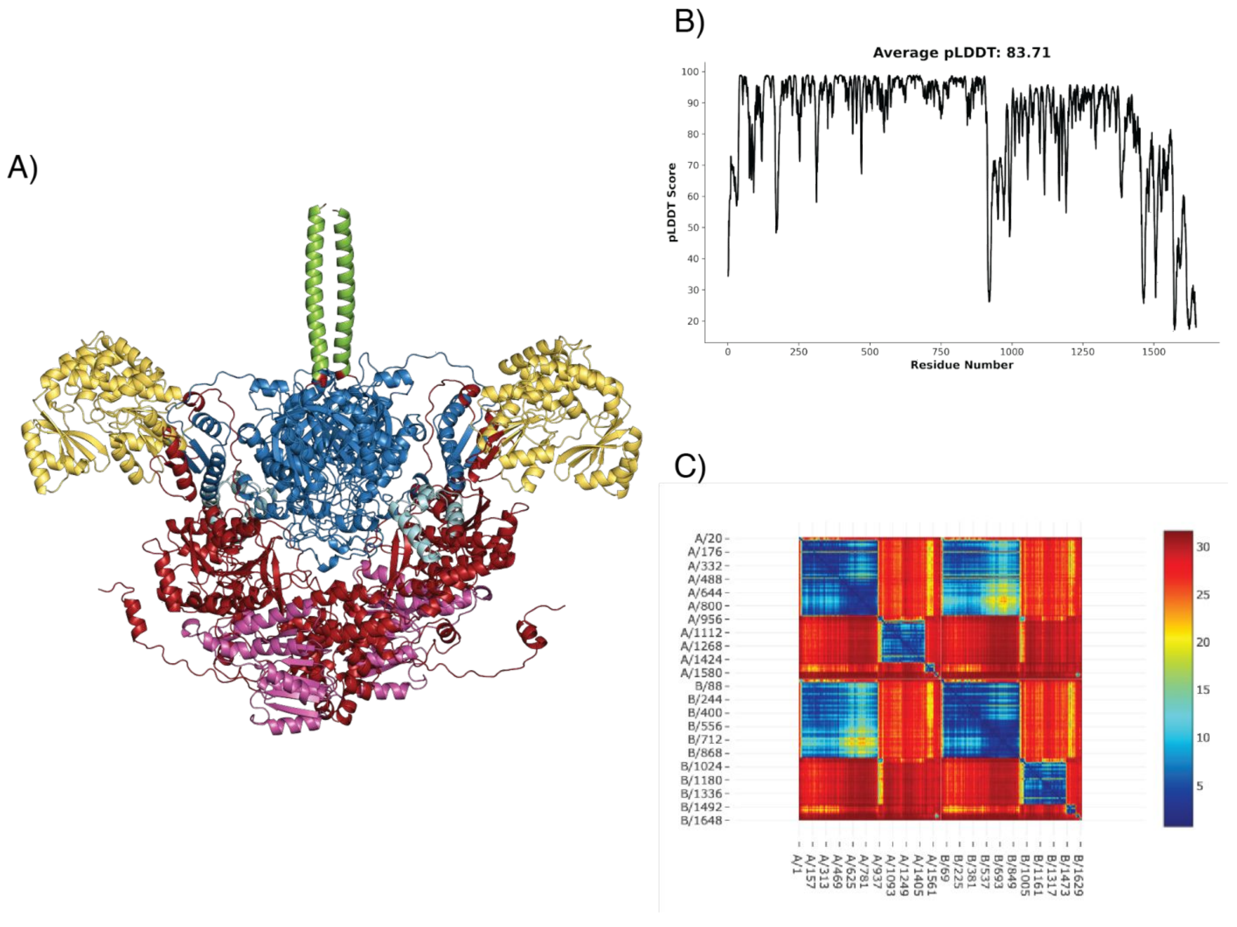

Figure S2. A) AlphaFold2 model of Lasalocid PKS Module 14. RMSD=1.97A with 7S6B. The domains are colored as follows: green = docking domains, blue = KS, yellow = AT, pink = KR, and light blue = ACP. B) AlphaFold2 pLDDT of model chain A by residue number C) AlphaFold2 predicted aligned error of model

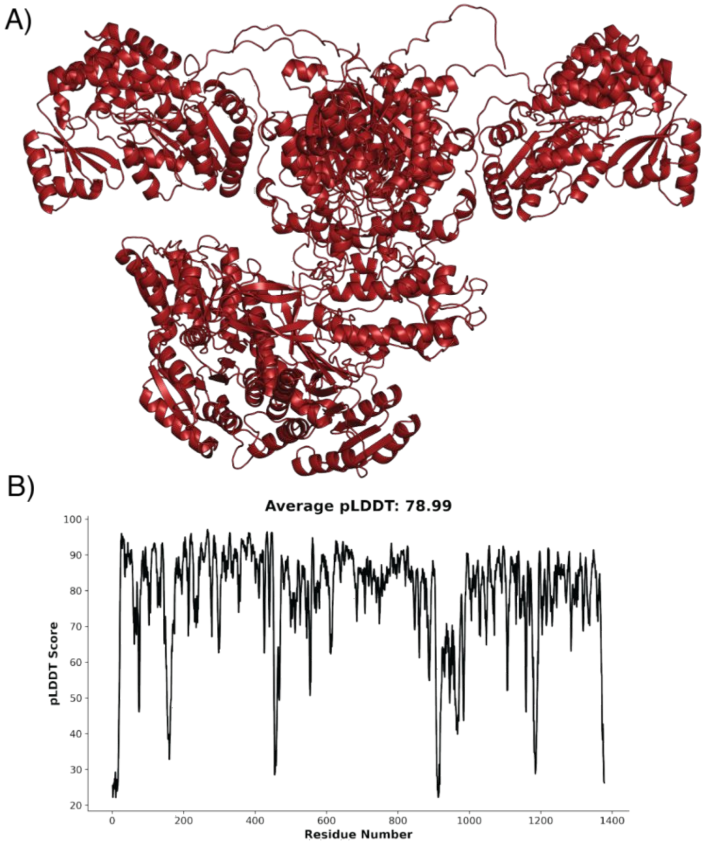

Figure S3. A) AlphaFold2 model of Lipomycin PKS Module 1 demonstrating asymmetric reaction chamber. B) AlphaFold2 pLDDT of model chain A by residue number

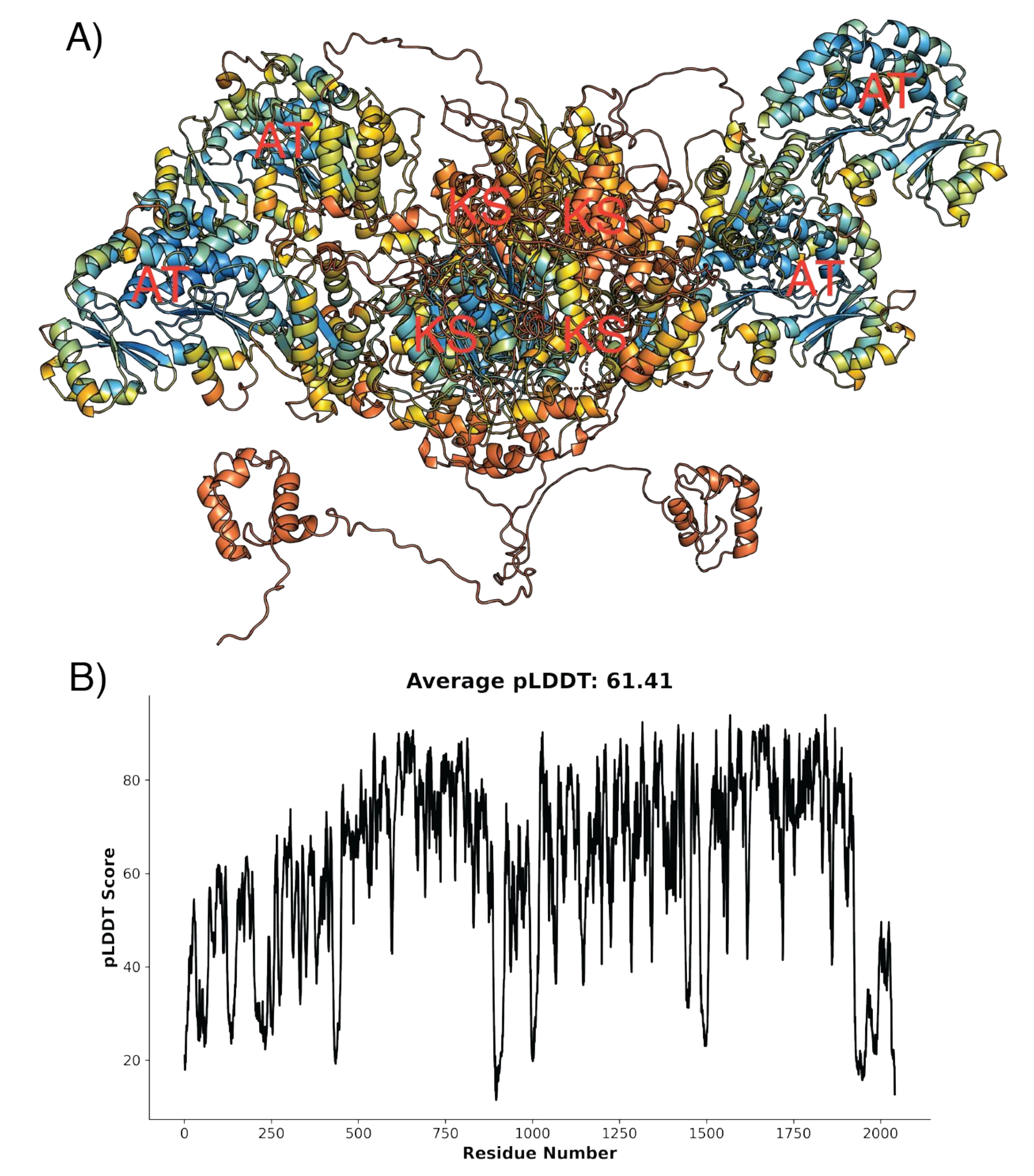

Figure S4. A) Attempt to model two consecutive PKS modules results in unreasonable steric artifacts. Shown is a PKS from *Streptomyces chumphonensis* that is bioinformatically predicted by antiSMASH to have a domain architecture of KS-AT-ACP-KS-AT-ACP colored by plldt. B) AlphaFold2 pLDDT of model chain A by residue number

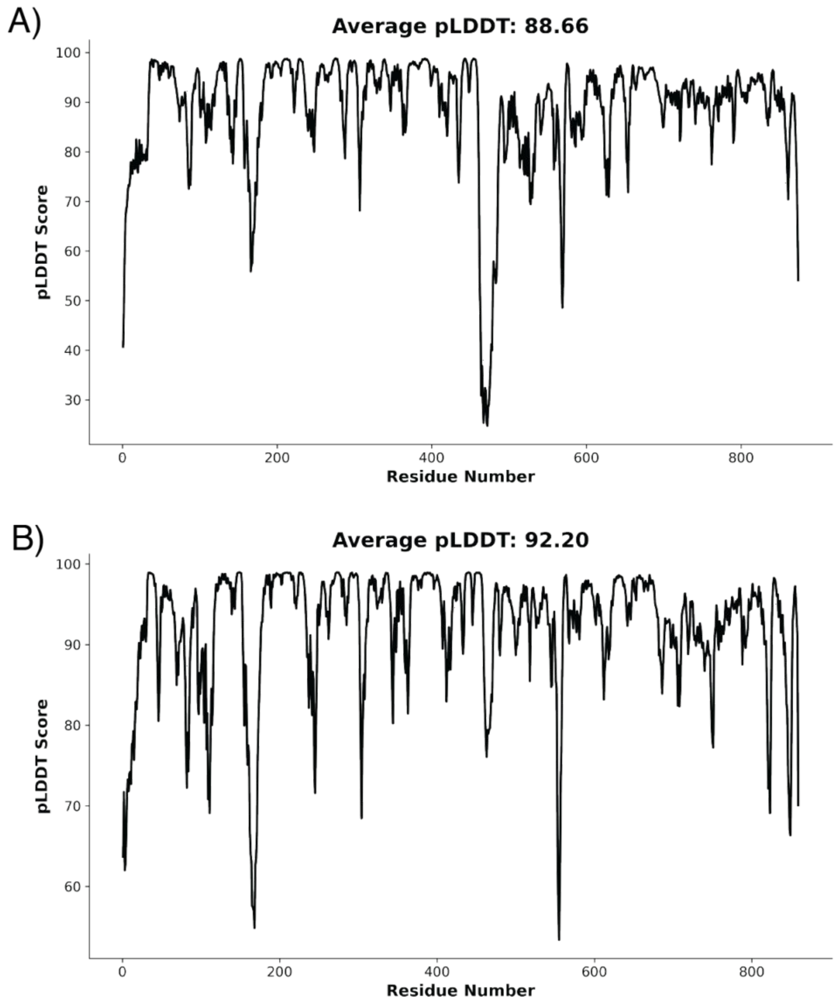

Figure S5. A) AlphaFold2 pLDDT of model chain A by residue number of Figure 3A lipM1 ACP with lipM2 KS-AT B) AlphaFold2 pLDDT of model chain A by residue number of Figure 3B epoM7 ACP with epoM7 KS-AT

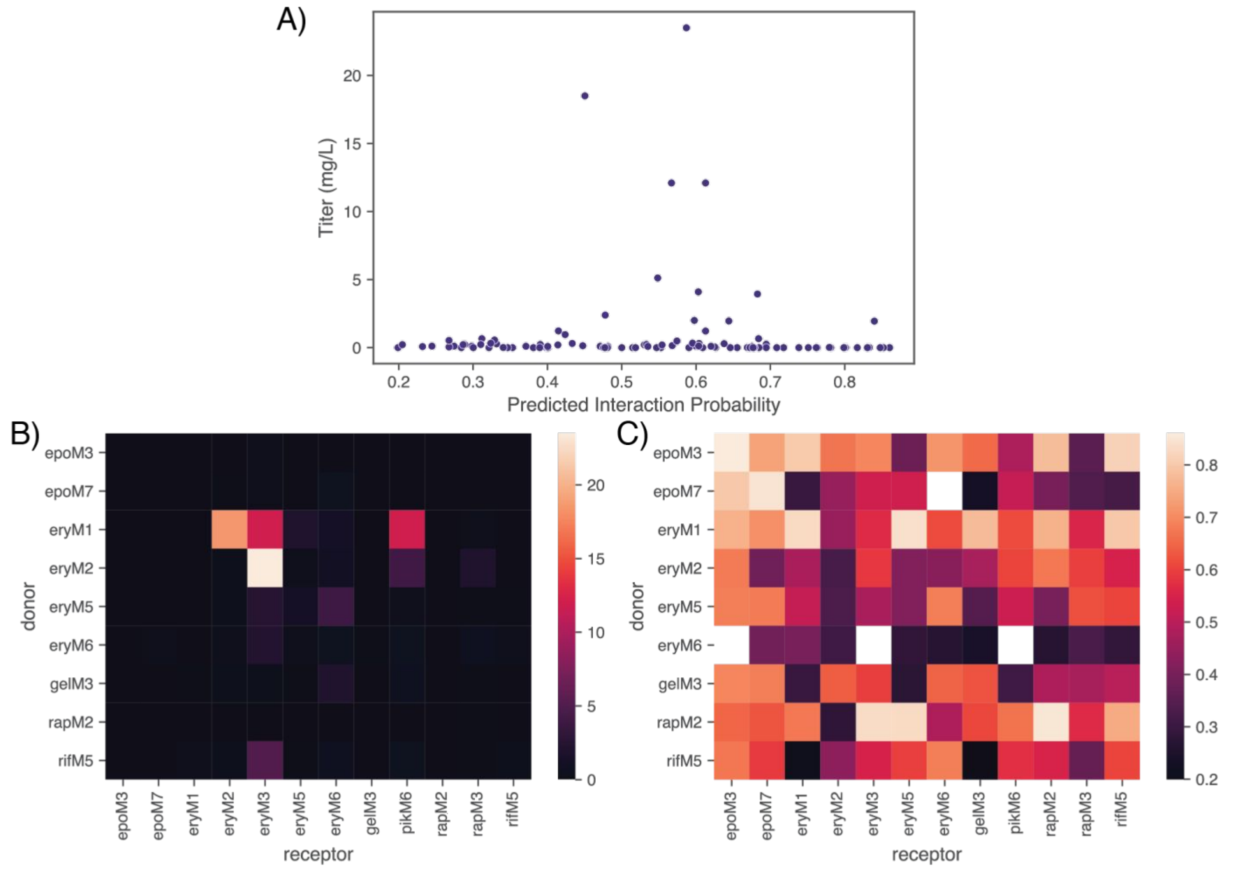

Figure S6. A) Predicted interaction probability between each upstream ACP (donor module ACP) and each downstream KS (acceptor module KS) and the observed production data from Menzella *et al.* B) Observed production titer (mg/L) of each combination. C) Predicted interaction probability of each combination.
